# Early Influence of the COVID‐19 Pandemic on Volunteer Water Monitoring Programs in the United States and Canada

**DOI:** 10.1111/1752-1688.13043

**Published:** 2022-07-09

**Authors:** Kristine F. Stepenuck, Jill Carr

**Affiliations:** ^1^ Rubenstein School of the Environment and Natural Resources, Lake Champlain Sea Grant and the Gund Institute for Environment The University of Vermont Burlington Vermont USA; ^2^ Massachusetts Bays National Estuary Partnership Boston Massachusetts USA

**Keywords:** volunteer monitoring, COVID‐19, coronavirus, pandemic, water monitoring, impacts

## Abstract

Volunteer water monitoring programs generate new scientific knowledge, contribute data to decision‐making processes, and increase social networks, technical knowledge, and skills of participants. Declaration of the COVID‐19 pandemic threatened the ability of these programs to continue to engage volunteers to achieve such outcomes. A national water monitoring network hosted a brainstorming webinar to facilitate communication across programs to identify potential solutions to pandemic‐influenced challenges. Following that webinar, a survey of United States and Canadian volunteer monitoring programs that was conducted about 3 months into the pandemic revealed that 72% of 80 responding programs planned to carry on through the 2020 field season despite most having experienced delayed starts. Other common program modifications implemented in the first months of the pandemic included adding COVID‐19 safety information to program guidance, changing field team composition, monitoring timing and logistics, and adopting new communications strategies. Most programs reported loss or anticipated loss in number of data observations (74%) and volunteers (66%), while 44% reported known or anticipated losses in funding. Seventeen percent of responding programs were able to swiftly develop distance learning tools to train participants, which led to increased program capacity to reach broader audiences.

## INTRODUCTION

Volunteer monitoring programs, particularly those focused on water resources and/or associated flora and fauna, have strong representation and ongoing contributions to data collection in the United States (U.S.) and Canada (Sharpe and Conrad 2006). In 2013, an estimated 1676 programs supported more than 100,000 individuals to participate each year in the U.S. (Stepenuck and Genskow 2018). In Canada, an increasing trend in community participation in volunteer water monitoring programs has been reported (Kebo and Bunch 2013). In Nova Scotia alone, at least 43 environmental stewardship programs that engage in water quality monitoring have collected data at hundreds of water bodies since the early 1990s (Sharpe and Conrad 2006). While volunteer water monitoring programs have varied monitoring schedules, many engage volunteers in monitoring multiple times throughout the year (Loperfido et al. 2010; Deutsch and Ruiz‐Córdova 2015). Furthermore, numerous U.S.‐based volunteer water monitoring programs have significant longevity with about 35% of 227 U.S.‐based programs reporting sustained monitoring for 20 or more years in a 2013 survey (Stepenuck and Genskow 2018).

The outcomes of these and other types of community science programs are numerous for the environment and for the individuals who participate (Stepenuck and Green 2015; Pocock et al. 2019). Engaging the public in the scientific process has been a useful tool in biodiversity conservation (Cooper et al. 2007), in producing data that are both scientifically valid and that address societal concerns (Jollymore et al. 2017), in contributing to natural resource policy and management decisions (Stepenuck and Genskow 2019), and in promoting conservation‐minded attitudes and actions (Jordan et al. 2011). Individuals who participate in community science programs are more likely to confidently participate in decision‐making processes (Ballard et al. 2008), and to learn technical and communication skills (Bela et al. 2016). Affectively, participation in volunteer environmental monitoring programs has allowed individuals to build social networks (Koss and Kingsley 2010), and has provided a meaningful way to connect with others (Lewandowski and Oberhauser 2017).

Volunteers are introduced to and engage in these monitoring programs in a variety of ways. They participate in trainings to learn methods (Kebo and Bunch 2013), and then follow defined methods to collect data to assess water quality and associated physical and biological parameters (e.g., aquatic life, habitat) in rivers and streams, lakes and ponds, at beaches, in wetlands, and in other types of waterbodies. In addition, volunteers often play supplemental roles to support their monitoring programs. These include selecting sites, analyzing data, and communicating results (Stepenuck and Genskow 2018).

When the COVID‐19 pandemic struck the world in March 2020, like other professionals, volunteer water monitoring program coordinators in the U.S. and Canada were caught off‐guard. For many, the timing of the pandemic and associated shutdowns coincided with traditional training periods and the start of the field season. Yet long‐term datasets, grant deliverables, and other outcomes such as enhanced social networks were at risk if monitoring programs were to cease for the year. Furthermore, spending time outdoors was recommended as a safe practice in which people could engage during the pandemic‐related shutdowns (Center for Disease Control, July 30, 2020. Deciding to Go Out. Accessed February 4, 2021. https://stacks.cdc.gov/view/cdc/91350/cdc_91350_DS1.pdf?). As a result, volunteer water monitoring program coordinators were eager to understand how they might move forward with their field seasons, affording volunteers a way to get outside and ensuring data were collected.

In response to receipt of numerous inquiries from program coordinators seeking knowledge of how other programs were adapting, a webinar was planned by the authors. The webinar was held in late April 2020 with the goal of identifying and sharing best practice recommendations for aquatic field sampling programs during COVID‐19 (USA Volunteer Monitoring Network. Volunteer Monitoring During COVID‐19. Accessed January 16, 2021. http://volunteermonitoring.org/covid). In total, 286 people registered for the webinar and 203 participated. More than 90 recommended best practices were generated through the webinar. These were summarized, and both the summary and a shared resource document were made available on the same website to allow for additional contributions as programs developed further guidance and materials during the 2020 field season.

This study was carried out in follow up to the initial April webinar to assess the impacts that the COVID‐19 pandemic had on volunteer water monitoring programs by early‐summer 2020. The study sought to understand the extent to which best practices identified through the webinar had been implemented, and to assess other types of outcomes that had resulted for volunteer water monitoring programs as a result of the pandemic.

## METHODS

A survey (approved via low‐risk exemption as STUDY00000950 by the University of Vermont, USA, Institutional Review Board, with anonymous analysis of data) to assess the impacts of the COVID‐19 pandemic on volunteer monitoring programs was conducted over an 8‐week period from late May to late July 2020 (see Supporting Information for complete survey and access to full results). Responses to the survey were sought from two groups: (1) individuals who participated in the April webinar who self‐identified as volunteer monitoring program coordinators/directors, staff, administrators, or volunteers; and (2) directors/coordinators of volunteer water monitoring programs included in a national directory available at http://volunteermonitoring.org whose programs were not represented in the April webinar. In total, 517 people were invited to participate in the survey, 286 who registered for the April webinar and 231 from the online program directory. To respect the stress of the pandemic on everyone and expectation that capacity would be limited to respond to a survey, each group received only an initial request and one reminder to complete the survey.

The survey assessed the impacts to volunteer monitoring programs broadly and identified the extent to which specific practices had been implemented. Broad level questions focused on understanding if there were expected negative impacts on the number of data observations, program funding, number of volunteers, or staff time in 2020. The survey also asked respondents to identify any possible benefits from the COVID‐19 pandemic on their volunteer monitoring programs. More specific questions focused on understanding if programs had implemented particular changes to protect volunteers and staff during the pandemic. In the development of the survey, 14 specific program changes were derived from the recommended best practices generated through the April webinar. Survey respondents were asked to identify if their volunteer monitoring programs had implemented any of the changes, and if they had not, to report the likelihood of implementing them in the near future. Accordingly, response options included “have implemented,” “likely to implement,” “unlikely to implement,” “definitely will not implement,” and “does not apply.” The types of program changes about which the survey inquired ranged from canceling monitoring in 2020 to more moderate program changes such as conducting training online, relying upon seasoned volunteers rather than recruiting new ones, and modifying sampling teams or methods. Follow‐up questions were posed to further explore potential programmatic impacts if a respondent indicated they had or intended to offer online training, or if they had or anticipated experiencing economic loss to their volunteer monitoring program.

## RESULTS

In total, 109 individuals submitted substantially complete responses to the survey, representing a 21% response rate. Three responses were removed prior to generating descriptive statistics; one was a duplicative response from a program director. The other two were from staff associated with a program for which a program director had also provided a response. As the study sought to understand program‐level impacts, only one response per program was desired. Seventy percent of respondents had participated in the April webinar or watched the recording. Respondents (*n* = 107) included program coordinators/directors (76%), program support staff (9%), program administrators (6%), volunteers (4%), and others not associated with a volunteer monitoring/community science program (6%). Respondents represented 34 U.S. states and four Canadian provinces.

**FIGURE 1:**
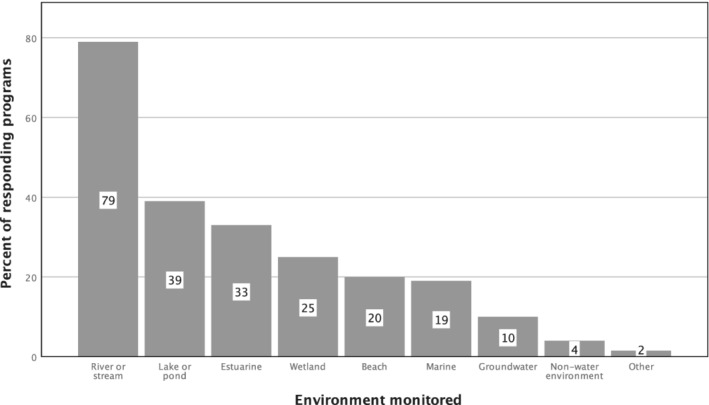
Types of environments monitored by percent of responding United States (U.S.) and Canadian volunteer monitoring program survey respondents (*n* = 107).

Programs monitored a variety of waterbody types (Figure 1), with rivers and stream monitoring programs most common (79%) and groundwater monitoring efforts least represented (10%). Other types of monitoring programs represented included those that assessed fish, macroinvertebrates, terrestrial animals (all reported by a single program), horseshoe crabs, submerged aquatic vegetation, and irrigation water. One program indicated training both field crews and volunteers. Numerous programs monitored more than one waterbody type, so percents do not add up to 100.

**FIGURE 2:**
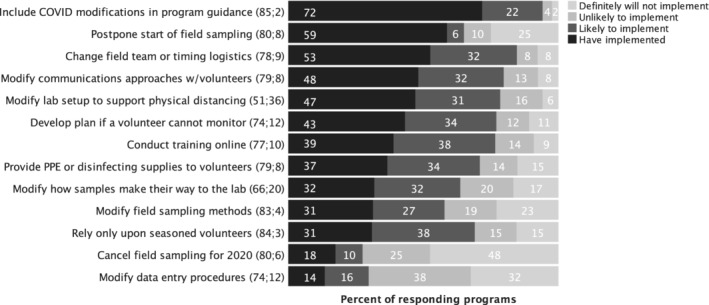
Changes made or anticipated in the first 3 months of the COVID‐19 pandemic by responding U.S. and Canadian volunteer monitoring programs. Numbers in parentheses are the number of responding programs to which the potential impact applied followed by the number of programs to which the potential impact did not apply. Percents displayed in bars represent only those programs to which the potential impact applied.

The majority (76%) expected to use or share knowledge gleaned from the webinar (*n* = 75), while 15% were unsure and the remaining 2% did not plan to use the information. Three types of program changes had been implemented by more than half of responding programs at the time the survey was conducted. The most common of these program changes, carried out by 72% of programs, was to include COVID‐19‐related safety guidance in program materials (Figure 2). This was followed closely by 59% of programs postponing the start of their annual field monitoring seasons. Another change that had already been implemented by over half of programs was timing of monitoring or field team composition to reduce inter‐household interactions (53%) with another 32% likely to implement this change. This included such modifications as having only household teams monitor together, staggering times of monitoring at a single site so that different households would not be present at the site at the same time, and/or assigning specific equipment or parameters to specific individuals to avoid the need for people to share equipment.

Three types of changes had been implemented by more than 40% but less than 50% of programs, and all three were likely to be implemented by about a third of responding programs to which the type of change was applicable. This included modifying communications approaches with volunteers (48%), modifying laboratory setups to accommodate for physical distancing (47%), and developing and communicating a plan if a volunteer was to become unable to monitor (43%).

On the other end of the scale, almost one in five (18%) programs had canceled their full 2020 field season. Conversely, 48% of programs indicated they definitely would not cancel their 2020 field season, and another 25% indicated they were unlikely to cancel, making this the most unlikely of modifications programs anticipated making.

For the 77% of programs that had already (39%) or were likely (38%) to offer training online, just less than half (44%) had or planned to offer their training live as opposed to directing volunteers to participate in a pre‐recorded asynchronous training (*n* = 59). However, the majority (63%) had or planned to use recorded videos to support the training they provided. Of those that planned to or had used videos (*n* = 37), 84% needed to create videos to use in their trainings. Almost one in five (19%) planned to or had used another program's training videos.

Other types of reported program modifications included offering trainings to individuals and developing online training modules, short courses or newsletter‐style trainings, requiring volunteers to commit to safety protocols in advance of training, and allowing volunteers to judge their own risk to determine whether or not to monitor. Others reported contracting with an external lab for macroinvertebrate identification, providing a full season of supplies to volunteers to avoid monthly equipment transfers, eliminating student staff, extending annual certifications, disinfecting all field equipment before distributing to volunteers, eliminating an advanced type of monitoring, and focusing on data analyses. One program reported making no modifications as volunteers monitored on their own lakeshore properties and communications had always been carried out without in‐person contact.

**FIGURE 3:**
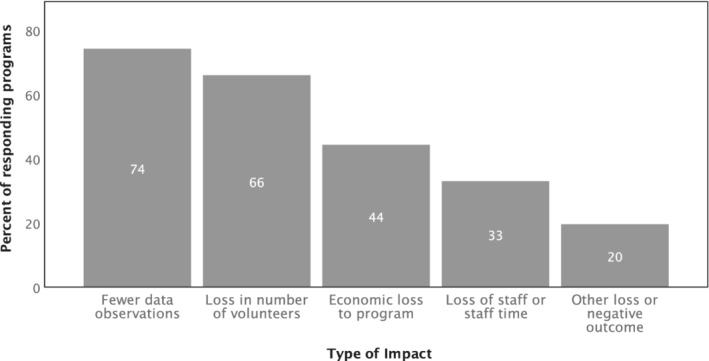
Types of impacts COVID‐19 had on responding U.S. and Canadian volunteer monitoring programs in the first 3 months of the COVID‐19 pandemic (*n* = 97).

Program staff members were asked about the types of losses their program had experienced or that they anticipated having as a result of COVID‐19. Almost three‐quarters of programs reported a loss in data observations (Figure 3). This may have related to loss in numbers of volunteers reported by two‐thirds of programs, and delays or cancellations in field seasons. A third of programs reported they had lost staff or staff time, while in open‐ended responses, three respondents noted that there were increased demands on staff and that work was taking longer due to pandemic‐related impacts.

Almost half of responding programs (44%) anticipated or had already faced economic losses (*n* = 43). The majority of those (58%) indicated that such losses would impact less than 25% of their annual incomes related to their community science program. About a third (34%) faced economic losses to 26–50% of their annual income. Relatively few (8%) faced economic losses to more than 50% of their annual program budgets. No responding program expected a 100% economic loss to their annual budget. Budgets ranged from less than $10,000/year (22%) to more than $150,000 (11%). About one in five programs (19%) reported annual budgets of $50,001–$100,000, while another 22% had budgets between $10,000 and $25,000, and the remaining 14% had budgets between $100,000 and $150,000.

**FIGURE 4:**
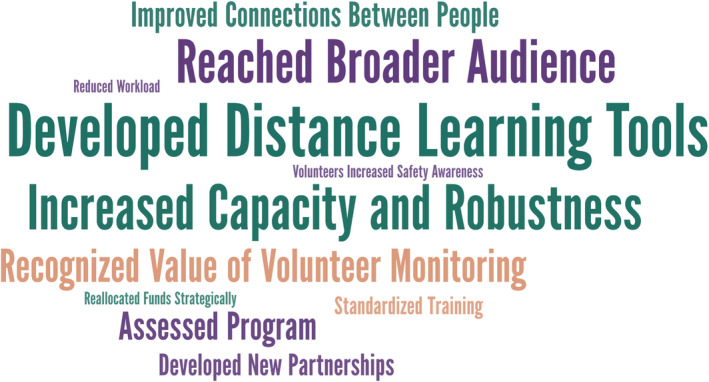
Positive outcomes of COVID‐19 on U.S. and Canadian volunteer monitoring programs in the first 3 months of the pandemic (*n* = 38). The larger the font, the more common the response. Phrases in the smallest font indicate a single response.

Respondents were also asked to identify if the COVID‐19 pandemic had resulted in any benefits for their program. Reported benefits were categorized by topic and displayed using a word cloud in which the size of the word indicates the frequency the topic was mentioned by respondents (Figure 4).

The most common benefit, mentioned by 18 programs (17%), was the development of distance‐learning tools. Related, 13 respondents (12%) recognized that development of such tools and other changes made to enable socially distanced interactions increased capacity and robustness of their programs. One person commented that, “it will increase our capacity against future disruptions.” Another noted that virtual meetings “may continue in addition to our usual face to face meetings so busy people can attend as travel and time permits.” Another commonly identified benefit, identified by 12 programs (11%), was that they had reached a broader audience. This happened in two ways. The first way was through increased volunteer recruitment, which one respondent theorized was driven by volunteers' interest to contribute to a valuable effort while spending time outside and with household members during the pandemic. The second way was through programs offering virtual trainings and educational events. This allowed an audience from a wider geographic area to participate and enabled those with other types of constraints that may have hindered in‐person attendance (e.g., children's bedtimes, vehicle access) to participate from wherever they were. One particularly compelling positive outcome that was reported related to recognition of the importance of volunteer monitoring to communities and to the development of scientific knowledge. For example, one coordinator shared that trained volunteers were able to continue monitoring while professional monitoring was canceled in 2020. This allowed a long‐term dataset to be enhanced and water quality conditions to be assessed throughout the summer season.

## DISCUSSION

Survey results of volunteer monitoring programs provided insight into the impacts of the pandemic on these programs during the first half of 2020. These observations provide a baseline for further research into pandemic‐related impacts and/or benefits to volunteer monitoring programs over time. Furthermore, this work can inform the broader population of volunteer environmental monitoring programs about the extent and types of impacts and modifications implemented by responding programs early in the pandemic. Key observations from the survey responses include that: (1) after a delayed start for most responding programs, volunteer monitoring was largely able to continue in 2020 with modifications to address pandemic‐related social distancing and other health guidelines; (2) the most common types of modifications implemented by responding programs to allow for monitoring to take place were shifts in field team composition and timing of monitoring, alterations to communications approaches with volunteers, and design and implementation of online trainings; (3) most programs reported a loss in number of data observations and volunteers during the 2020 field season; and (4) the most commonly reported positive outcome resulting from the pandemic by June 2020 was development of distance learning tools, which, in turn, were often predicted to provide programs with increased capacity and/or opportunity to reach an expanded audience.

It is notable that the majority of volunteer monitoring programs had been able to or anticipated being able to continue field‐monitoring operations in 2020. Each had worked or anticipated working successfully through a situation that required significant planning, reorganization, and swift action to address. This is a reflection of the existing structures and procedures of these programs, and of the capacity of program staff to respond efficiently to a rapidly changing situation. Such a response speaks to the organizational resilience of these programs. Important to note, though, is that such an intensive pivot in communication and implementation strategies may have led to program coordinator burnout, the effects of which may not have been captured given the timing of our survey early in the pandemic.

Resilience has been defined as “the ability to recognize and adapt to handle unanticipated perturbations … and demand a shift of processes, strategies, and coordination” (Woods 2012, 22). A variety of literature delineates components of, and influences on, organizational resilience, including organizational structure and culture (Sawalha 2015), leadership (Carpenter et al. 2012), capacity to adapt (Lee et al. 2013), and the ability to learn (Walker 2020) and to apply new ideas and processes (Tyler and Moench 2012). While the former two characteristics may have played a role in the ability of programs to respond to the pandemic, these were not measured through the survey, and might be explored in future research. Evidence does exist to support that programs were effectively able to swiftly adapt, learn, and apply new ideas in response to the pandemic. For instance, programs implemented a variety of physical distancing procedures — such as defining ingress and egress pathways at laboratories, shifting mechanisms for delivery and pick up of equipment and samples, and changing field team composition, responsibilities, and timing. The majority of programs also changed or expected to change how they provided trainings and the manner in which they communicated with volunteers. Similarly, the most commonly reported positive outcome of the pandemic for the programs was to have developed distance learning tools.

Increased resilience can also be achieved when organizations have access to information necessary to address challenges at hand (Tyler and Moench 2012). The network of volunteer monitoring programs in North America served as information brokers to one another by seeking (through informal contacts known through past networking opportunities) and then sharing pandemic‐related modification suggestions with one another through the April webinar. 76% of programs had already used or were planning to use information from the webinar in the following 6–12 months, and 19% of programs that had shifted to offering remote trainings had used videos from other programs. The national network of volunteer monitoring programs has existed in some capacity since the early 1990s when the US Environmental Protection Agency supported development of shared methods, a series of national conferences, and a national directory of programs (USEPA, EPA's Volunteer Monitoring Program. Accessed February 4, 2021. https://archive.epa.gov/water/archive/web/html/epasvmp.html), along with a national newsletter that was produced for 17 years, ending production in 2010 (E. Ely, The Volunteer Monitor Project. Accessed February 4, 2021. https://archive.epa.gov/water/archive/web/html/issues.html). In the past two decades, Extension (USA Volunteer Monitoring Network. Accessed February 4, 2021. http://volunteermonitoring.org), the National Water Quality Monitoring Council (National Water Quality Monitoring Council. Volunteer Monitoring. Accessed February 4, 2021. https://acwi.gov/monitoring/vm/index.html), the Citizen Science Association (Citizen Science Association. Accessed February 4, 2021. https://citizenscience.org/), NOAA Offices of Education and Fisheries (NOAA Office of Education. Citizen Science and Crowdsourcing. Accessed June 10, 2022. https://www.noaa.gov/office‐education/citizen‐science‐crowdsourcing) and Sea Grant (NOAA Sea Grant Citizen Science Network Vision. Accessed February 4, 2021. https://seagrant.noaa.gov/Portals/1/CommunityScience‐NetworkVision2018‐2.pdf) have all played roles in continuing to support national and international listservs, conferences, workshops and webinars to support the network. Opportunity exists for these and other similar networks to continue to offer professional development training to address identified needs of the volunteer monitoring programs during (or following) the pandemic. For programs responding to this survey, that includes providing guidance, tools, and resources to aid programs in developing video trainings and to effectively lead remote trainings.

Resilience has limits, however. The size of a disruption to programming, nimbleness of a program to respond to the challenge presented, and capacity to meet other programmatic demands all play a role in the ability of a program to be resilient (Woods 2012). Disparate disruptions may affect various aspects of these programs. For instance, as new COVID‐19 variants continue to emerge, volunteer participation may diminish due to the vulnerability of the core of many programs' volunteer workforce — that is, older individuals (Jones et al. 2018; Füchslin et al. 2019). Vaccine and booster availability and efficacy may further affect volunteer willingness to participate. Furthermore, supply chain issues may influence the completeness of datasets if monitoring equipment is unavailable, and with compromised data completeness, data usability, and perceived program value may decline. In addition, reductions in state or federal budgets (Auerbach and Gale 2020), and/or nongovernmental funding sources may reduce the ability of these programs to support data collection or volunteer training and support. While such outcomes may result, the widely varied social responses to the pandemic and varied timing and intensity of infection across geographic areas may result in distinctly unique scenarios playing out in varied locations. Each program will need to assess available funding, staff, volunteers, and resources to determine if required deliverables can be met. The 18% of programs that canceled their 2020 field seasons may be more vulnerable to further impacts in the coming years, especially if their field seasons are repeatedly impacted. Strategies that coordinators might implement to maintain a program with reduced funds or manpower might include paring down the number of sampling sites to target only those of highest priority (e.g., water quality hot spots), reducing the number of costly lab samples (e.g., analyze nutrients once a month instead of biweekly), and taking advantage of no‐cost equipment loan programs offered through many state and federal governments. In addition, programs might seek new partnerships with municipalities and other environmental organizations within and adjacent to their watershed to find efficiencies, given that they may share monitoring goals.

The loss in data observations reported by two‐thirds of programs has potential to impact individual programs in myriad ways. The level of impact will depend on the program goals and objectives, parameters monitored, the timing and frequency of monitoring, and the level of completeness of datasets required. Programs may lose the ability to track seasonal changes in parameters monitored (e.g., such as where spring and fall macroinvertebrate monitoring is recommended due to expected differences) if the spring season was missed due to delayed start to field monitoring. Other programs with educational objectives may experience impacts to student learning through reduced opportunity for them to use standardized equipment and methods to collect data or conduct laboratory analyses. Still, others may face cascading impacts to monitoring in future years due to data losses in 2020. For instance, this may occur where monthly samples are required by state guidelines to determine if a waterbody is impaired, and data collection was impeded for one or more months.

Two‐thirds of programs reported loss in number of volunteers in 2020. This may have been due in part to programs limiting participation to seasoned volunteers only, as 31% of programs had done by the time the survey was conducted. Nearly 40% more programs anticipated limiting participation during the 2020 field season. Volunteer participation may also have been limited due to personal health concerns or pandemic‐related restrictions on travel or gathering. Like loss in data collected, impacts of loss of volunteers on program function and success are anticipated to vary greatly across programs.

If conditions allow for new participant recruitment in future years (including having sufficient funding to support staff to recruit and manage volunteers), programs may easily rebound to pre‐pandemic levels of participation. During early months of the pandemic, outdoor recreation increased in neighborhoods (Rice et al. 2020), and in urban green spaces, particularly those that were more remote, suggesting people had ventured to those areas to find solace and safe space (Venter et al. 2020). As volunteer monitoring gets people outdoors and often off the beaten path to reach monitoring sites, such findings suggest that participation in volunteer monitoring could be boosted as a result of the pandemic if programs are able to safely recruit and train participants. In fact, increased interest by community members to participate was observed by some of the responding programs during the survey period. The motivation for volunteers to continue to participate during the COVID‐19 pandemic was also observed in southern Africa where volunteer data contributions to a bird monitoring project declined by only 15%, as compared to 70% decline in overall data contributions (by professionals and volunteers) in the broader program (Rose et al. 2020). This was attributed both to volunteer participants having an interest in nature and wishing to contribute to the scientific endeavor (Rose et al. 2020). A recent study of U.S.‐based volunteer water quality monitoring programs found that the two strongest drivers of participation were to benefit the environment and aid in generation of scientific knowledge (Alender 2016). These align with findings from other organizations with conservation missions (Bruyere and Rappe 2007), and other community science programs (Hobbs and White 2012; Domroese and Johnson 2016). In addition, having the opportunity to be in nature was identified as a critical factor for participation in a Canadian community science program (Ng et al. 2018).

Alternatively, focused retention strategies may be particularly important for programs to implement if pandemic‐related social distancing restrictions continue for an extended period beyond 2020, and programs continue to rely upon seasoned volunteers. Nurturing existing volunteers to encourage longer‐term participation may aid programs in achieving desired goals, including conservation outcomes (Beirne and Lambin 2013). Best practices for volunteer retention include providing ongoing educational opportunities and networking (Skoglund 2006), regularly communicating with volunteers (West and Pateman 2016), and acknowledging volunteer contributions (Wolcott et al. 2008; Walk et al. 2019). In 2021 and in years to come, programs have the opportunity to use refined communications techniques and newly found distance education skills and tools developed in 2020 to afford volunteers continuing education as well as networking opportunities. In survey responses, some programs noted the opportunity to build connections among participants as a benefit from the pandemic. Continued sharing of knowledge and resources among programs across North America and beyond in the coming years could further enhance the ability of programs to sustain volunteer participation. Opportunity exists to develop educational programs and videos to enhance volunteer learning that could be shared widely. Such educational initiatives might also serve as a mechanism to build volunteer networks not only within programs, but across the broader network of North American volunteer monitoring programs. Existing networks might serve as catalysts to establish not only program coordinator‐focused trainings, but those that address interests of volunteers, or as clearinghouses for recorded educational programs. These networks might also serve to promote recognition of volunteers for their continued dedication to understanding their local water and associated natural resources.

There were several study limitations including that the survey respondents self‐selected to respond, and thus may have had more capacity or lesser impacts of the pandemic than non‐responding programs. For instance, some programs may have lost funding during spring 2020 and staff may not have been on board when the survey was distributed. Others may have had limited staff without capacity to respond to a voluntary survey about their current situation. Another limitation of this study is that results cannot be generalized to the broader population of volunteer monitoring programs in North America as a random sample of volunteer monitoring programs was not surveyed. A random sample would enable less biased tracking of pandemic‐induced impacts on, resources developed for, and modifications made to programs over time.

Additionally, individual monitoring programs should evaluate program success in light of a continually evolving pandemic. Assessment of success could be based on annual metrics such as the retention rates and performance evaluations of volunteers trained virtually vs. in‐person. Furthermore, the program's sampling completeness (e.g., percentage of planned sites and parameters that were actually sampled), amount of funding secured, and proportion of deliverables met (e.g., trainings, annual reports, public engagement programs) could gauge program success. Future research could evaluate these same success metrics over time, comparing pre‐pandemic results (when available) to those collected at various times as the pandemic progresses to further quantify the influence of COVID‐19 on monitoring programs.

## CONCLUSIONS

The COVID‐19 pandemic has changed the world, yet the majority of U.S. and Canadian volunteer monitoring programs that responded to an early summer 2020 survey to assess modifications made and impacts to programs revealed that the majority had a 2020 field season despite most having delayed starts. Modifications to program guidance documents, field team composition, monitoring timing and logistics, communications, trainings, equipment distribution, sample delivery, and laboratory set up were commonly reported. Most programs reported loss or anticipated loss in data observations and volunteers, while just less than half of programs reported economic losses as a result of the pandemic. Positive outcomes reported included development of distance learning tools, opportunity to reach a broader audience due to new communications strategies and increased program capacity. In upcoming field seasons, programs must take stock of budgets, staff, volunteers, and other resources, and implement actions to retain volunteers over time or to recruit and train volunteers to sustain programs and data collection over time. Opportunities exist for national volunteer monitoring, and community and citizen science networks to provide or support shared distance learning among programs to afford professional development to staff and volunteers, to strengthen volunteer networks using recently learned communication technologies, and to recognize volunteer contributions broadly.

## AUTHOR CONTRIBUTIONS

**Kristine F. Stepenuck**: Conceptualization; data curation; formal analysis; investigation; methodology; project administration; supervision; visualization; writing – original draft. **Jill Carr**: Conceptualization; methodology; project administration; supervision; writing – review and editing.

## Supporting information

The full survey and results.

## Data Availability

The data that support the findings of this study are available in Supporting Information of this article.
