# Supplementary material for: Early Influence of the COVID‐19 Pandemic on Volunteer Water Monitoring Programs in the United States and Canada
Source: J Am Water Resour Assoc. 2022 Jul 9:10.1111/1752-1688.13043. Online ahead of print. doi: 10.1111/1752-1688.13043 (PMC9349743; doi:10.1111/1752-1688.13043)
Supplement: Supplementary file 1 — The full survey and results. [file JAWR-9999-0-s001.zip › jawr13043-sup-0002-SupinfoS2.docx]

The survey that was implemented during spring and early summer 2020 to U.S. and Canadian-based volunteer water monitoring programs follows. Logic used to display certain questions based on responses to earlier questions in the survey is included in grey or blue highlighted areas of each page. Anonymized results are available as a separate supporting document.

COVID and Volunteer Monitoring

Start of Block: Default Question Block

Q1 **Research Information Sheet**    Title of Study: COVID Impacts on Volunteer Water Monitoring and Citizen Science Programs   Principal Investigator (PI): Dr. Kristine Stepenuck                  Funder: NOAA National Sea Grant Program   **Introduction** Thank you for your participation in or interest in the *Citizen-based aquatic field sampling in the time of COVID*webinar that was held on [April 21, 2020](http://volunteermonitoring.org/covid). You are being invited to participate in a research study due to your participation in the webinar or your interest/participation in aquatic citizen-based/volunteer monitoring.    **Purpose** This research is intended to help understand the extent to which ideas that were shared during that meeting have been or may be implemented, as well as to understand ongoing programmatic needs and impacts related to volunteer water monitoring and COVID-19, and to define next steps for shared learning. This study is being conducted by Dr. Kristine Stepenuck at the University of Vermont. You must be at least 18 years of age to participate.   **Study Procedures** If you choose to participate, you will be asked to complete an online survey that will take about 10 minutes of your time. You will be asked questions about your intended uses of information shared during the meeting, potential for the ideas shared to be implemented by volunteer water monitoring programs, impacts the COVID-19 situation has had or is expected to have on volunteer monitoring programs, and ongoing information needs to aid in program response to the COVID-19 situation.    **Benefits** As a participant, there may not be any direct benefit for you; however, information from this study may benefit other people now or in the future who lead or participate in volunteer water monitoring or citizen science programs as future meetings will be developed based on results.   **Risks**   We do not anticipate there being risk to you to participate in this study. We will not collect any information that will identify you to protect your confidentiality   **Costs** There will be no costs to you for participation, and you will not be paid for taking part in this study.   **Compensation** You will not be paid for taking part in this study.   **Confidentiality** All information collected about you during the course of this study will be stored with a code name or number so that we are able to match you to your answers.      Survey results will be stored on a password-protected computer of the principal investigator and password-protected cloud software used to collect survey results. Results and recommendations are expected to inform upcoming presentations and meetings, and be shared in a peer-reviewed journal article, and on volunteer monitoring/citizen science websites and/or social media. While volunteer monitoring or citizen science program name will be collected during this study, that information will be used only to determine duplicate responses when tallying results (e.g., if multiple staff respond from a single program). This information will be coded to identify multiple responses from a single program, and original results will be deleted. All results to be shared in presentations, papers, websites or otherwise will be anonymous.   **Voluntary Participation/Withdrawal** Taking part in this study is voluntary. You may choose not to take part, not to answer any question, or to withdraw at any time. If you decide to take part, you can change your mind later and withdraw from the study. In that case, any answers you submitted will be deleted from the master dataset.

Questions If you have any questions about this study now or in the future, you may contact Dr. Kristine Stepenuck at: 802-868-1048. If you have questions or concerns about your rights as a research participant, then you may contact the Director of the University of Vermont Research Protections Office at (802) 656-5040.    If you are willing to participate, please click on arrows at the bottom of each screen to move through the survey.   It is recommended you print this information sheet for your records before continuing.

|  |  |
| --- | --- |

Q2 Did you participate in the *Citizen-based aquatic field sampling in the time of COVID* webinar on April 21, 2020?

- Yes (1)
- No, but I watched the recording or reviewed available online materials (2)
- No (3)

Skip To: Q5 If Did you participate in the Citizen-based aquatic field sampling in the time of COVID webinar on A... = No

| Page Break |  |
| --- | --- |

Display This Question:

If Did you participate in the Citizen-based aquatic field sampling in the time of COVID webinar on A... = Yes

Or Did you participate in the Citizen-based aquatic field sampling in the time of COVID webinar on A... = No, but I watched the recording or reviewed available online materials

Q3 Do you expect to use or share any knowledge or practices you learned from the webinar in the next 6-12 months?

- Yes (1)
- No (2)
- Unsure (3)
- NA (4)

| Page Break |  |
| --- | --- |

Display This Question:

If Do you expect to use or share any knowledge or practices you learned from the webinar in the next... = Yes

Q4 In one or two sentences, please briefly describe ***what information you learned*** from the webinar ***and how you plan to use or share that information*** within the next 6-12 months.

________________________________________________________________

________________________________________________________________

________________________________________________________________

________________________________________________________________

________________________________________________________________

| Page Break |  |
| --- | --- |

Q5 Please identify your role within the field of volunteer water monitoring/citizen science. Check the answer that best fits your role.

- Program Coordinator/Director (1)
- Program support staff (2)
- Program administrator (3)
- Volunteer monitor (4)
- Not associated with a specific volunteer monitoring or citizen science program (please describe your role) (5) ________________________________________________

| Page Break |  |
| --- | --- |

Display This Question:

If Please identify your role within the field of volunteer water monitoring/citizen science. Check t... = Program Coordinator/Director

Or Please identify your role within the field of volunteer water monitoring/citizen science. Check t... = Program support staff

Or Please identify your role within the field of volunteer water monitoring/citizen science. Check t... = Program administrator

Or Please identify your role within the field of volunteer water monitoring/citizen science. Check t... = Volunteer monitor

Q6 What type of environments are monitored in your volunteer water monitoring or citizen science program? Check all that apply.

- Marine (1)
- Estuarine (2)
- Lake or pond (3)
- River or stream (4)
- Beach (5)
- Wetland (6)
- Groundwater (7)
- Other type of environment, phenomenon, animal, etc. (i.e., something not related to water) (please describe) (8) ________________________________________________
- Other (please describe) (9) ________________________________________________

| Page Break |  |
| --- | --- |

Display This Question:

If Please identify your role within the field of volunteer water monitoring/citizen science. Check t... = Program Coordinator/Director

Or Please identify your role within the field of volunteer water monitoring/citizen science. Check t... = Program support staff

Or Please identify your role within the field of volunteer water monitoring/citizen science. Check t... = Program administrator

Q7 Please provide the name of the volunteer water monitoring program and organization with which you are associated. This will allow duplicate responses from a single program to be combined when summarizing data. Neither program names nor organizations will be shared in any summary reports, follow up meetings or otherwise.

________________________________________________________________

| Page Break |  |
| --- | --- |

Display This Question:

If Please identify your role within the field of volunteer water monitoring/citizen science. Check t... = Program Coordinator/Director

Or Please identify your role within the field of volunteer water monitoring/citizen science. Check t... = Program support staff

Or Please identify your role within the field of volunteer water monitoring/citizen science. Check t... = Program administrator

Q9 To assess changes volunteer water monitoring or citizen science programs have implemented or intend to implement as a result of COVID-19, for each of the following, please identify how likely your program is to do each of the following:

|  | Have implemented (1) | Likely to implement (2) | Unlikely to implement (3) | Definitely will not implement (4) | Does not apply (5) |
| --- | --- | --- | --- | --- | --- |
| Cancel field sampling for 2020 (1) |  |  |  |  |  |
| Postpone the start of field sampling (2) |  |  |  |  |  |
| Update program guidance to include COVID-related program modifications (3) |  |  |  |  |  |
| Conduct training online (4) |  |  |  |  |  |
| Rely only upon seasoned volunteers (i.e., no new volunteers this year) (5) |  |  |  |  |  |
| Change field team or timing logistics (e.g., solo or household teams, staggered times, specific equipment assignments) (6) |  |  |  |  |  |
| Provide PPE or cleaning/disinfecting supplies to volunteers (7) |  |  |  |  |  |
| Modify field sampling methods (e.g., add or remove certain types of sampling) (8) |  |  |  |  |  |
| Modify data entry procedures (9) |  |  |  |  |  |
| Modify how samples make their way to the lab (10) |  |  |  |  |  |
| Modify lab setup to support physical distancing (e.g., install partitions, one-way ingress-egress) (11) |  |  |  |  |  |
| Develop and communicate a plan for if a volunteer cannot monitor (12) |  |  |  |  |  |
| Modify communications approaches with volunteers (e.g., facilitate online meet-ups or social media sharing among volunteers; offer online question/answer sessions) (13) |  |  |  |  |  |
| Other (please describe) (14) |  |  |  |  |  |

| Page Break |  |
| --- | --- |

Display This Question:

If To assess changes volunteer water monitoring or citizen science programs have implemented or inte... = Conduct training online [ Have implemented ]

Or To assess changes volunteer water monitoring or citizen science programs have implemented or inte... = Conduct training online [ Likely to implement ]

Q10 Please describe how you plan to or have provided your online training.

|  | Yes (1) | No (2) | Does not apply (3) | Unknown / TBD (4) |
| --- | --- | --- | --- | --- |
| Was/Will your training (be) offered live? (1) |  |  |  |  |
| Did you/will you use recorded videos? (2) |  |  |  |  |

| Page Break |  |
| --- | --- |

Display This Question:

If Please describe how you plan to or have provided your online training. = Did you/will you use recorded videos? [ Yes ]

Q11 Did you need to create new training videos or did they already exist? Check all that apply.

- Existed for my program (1)
- Need(ed) to create (2)
- Used/will use videos from another organization (3)
- Other (please describe) (4) ________________________________________________

| Page Break |  |
| --- | --- |

Display This Question:

If Please identify your role within the field of volunteer water monitoring/citizen science. Check t... = Program Coordinator/Director

Or Please identify your role within the field of volunteer water monitoring/citizen science. Check t... = Program support staff

Or Please identify your role within the field of volunteer water monitoring/citizen science. Check t... = Program administrator

Q12 Please feel free to share other actions not previously mentioned that you are planning to implement, or that you have implemented to protect volunteers and/or staff during the pandemic.

________________________________________________________________

________________________________________________________________

________________________________________________________________

________________________________________________________________

________________________________________________________________

| Page Break |  |
| --- | --- |

Display This Question:

If Please identify your role within the field of volunteer water monitoring/citizen science. Check t... = Program Coordinator/Director

Or Please identify your role within the field of volunteer water monitoring/citizen science. Check t... = Program support staff

Or Please identify your role within the field of volunteer water monitoring/citizen science. Check t... = Program administrator

Q13 Do you anticipate any of the following losses as a result of COVID-19-induced programmatic changes? Please check all that apply.

- Reduction in number of data observations (1)
- Economic loss to your volunteer monitoring program (2)
- Loss in number of volunteers (3)
- Loss of staff or staff time (4)
- Other type of loss or negative outcome (please describe) (5) ________________________________________________

| Page Break |  |
| --- | --- |

Display This Question:

If Do you anticipate any of the following losses as a result of COVID-19-induced programmatic change... = Economic loss to your volunteer monitoring program

Q14 What is the extent of expected economic loss to your volunteer monitoring or citizen science program? (If you have a broader organization than just volunteer water monitoring and can report on just the volunteer monitoring program component, that’s what we’re aiming to collect here and in the next question.)

- <25% (1)
- 26-50% annual income (2)
- 51-75% annual income (3)
- 76-99% annual income (4)
- 100% annual income (5)

Display This Question:

If Do you anticipate any of the following losses as a result of COVID-19-induced programmatic change... = Economic loss to your volunteer monitoring program

Q15 In 2019, in what range was your annual volunteer monitoring program income?

- <$10,000 (1)
- $10,001-$25,000 (2)
- $25,001 - $50,000 (3)
- $50,001 – $100,000 (4)
- $100,001 - $150,000 (5)
- >$150,000 (6)

| Page Break |  |
| --- | --- |

Display This Question:

If Please identify your role within the field of volunteer water monitoring/citizen science. Check t... = Program Coordinator/Director

Or Please identify your role within the field of volunteer water monitoring/citizen science. Check t... = Program support staff

Or Please identify your role within the field of volunteer water monitoring/citizen science. Check t... = Program administrator

Q16 Please list any known or anticipated benefits for your volunteer water monitoring or citizen science program as a result of the COVID-19 pandemic.

________________________________________________________________

________________________________________________________________

________________________________________________________________

________________________________________________________________

________________________________________________________________

| Page Break |  |
| --- | --- |

Display This Question:

If Please identify your role within the field of volunteer water monitoring/citizen science. Check t... = Program Coordinator/Director

Or Please identify your role within the field of volunteer water monitoring/citizen science. Check t... = Program support staff

Or Please identify your role within the field of volunteer water monitoring/citizen science. Check t... = Program administrator

Or Please identify your role within the field of volunteer water monitoring/citizen science. Check t... = Volunteer monitor

Q17 In what state/province (as applicable) and country is your volunteer water monitoring or citizen science program based?

________________________________________________________________

| Page Break |  |
| --- | --- |

Q18 What information needs do you have related to COVID-19 that could be addressed in future webinars?

________________________________________________________________

________________________________________________________________

________________________________________________________________

________________________________________________________________

________________________________________________________________

| Page Break |  |
| --- | --- |

Q19 Please share any other comments you have below.

________________________________________________________________

________________________________________________________________

________________________________________________________________

________________________________________________________________

________________________________________________________________

End of Block: Default Question Block
